# Preconceptual Priming Overrides Susceptibility to Escherichia coli Systemic Infection during Pregnancy

**DOI:** 10.1128/mBio.00002-21

**Published:** 2021-02-23

**Authors:** Nina Salinger Prasanphanich, Emily J. Gregory, John J. Erickson, Hilary Miller-Handley, Jeremy M. Kinder, Sing Sing Way

**Affiliations:** a Division of Infectious Diseases, Center for Inflammation and Tolerance, Cincinnati Children’s Hospital Medical Center, University of Cincinnati College of Medicine, Cincinnati, Ohio, USA; b Department of Obstetrics and Gynecology, Cincinnati Children’s Hospital Medical Center, University of Cincinnati College of Medicine, Cincinnati, Ohio, USA; c Division of Neonatology, Cincinnati Children’s Hospital Medical Center, University of Cincinnati College of Medicine, Cincinnati, Ohio, USA; University of Illinois at Chicago

**Keywords:** *Escherichia coli*, pregnancy, prenatal infection, preconceptual, vaccination

## Abstract

Maternal sepsis is a leading cause of morbidity and mortality during pregnancy. Escherichia coli is a primary cause of bacteremia in women and occurs more frequently during pregnancy. Several key outstanding questions remain regarding how to identify women at highest infection risk and how to boost immunity against E. coli infection during pregnancy. Here, we show that pregnancy-induced susceptibility to E. coli systemic infection extends to rodents as a model of human infection. Mice infected during pregnancy contain >100-fold-more recoverable bacteria in target tissues than nonpregnant controls. Infection leads to near complete fetal wastage that parallels placental plus congenital fetal invasion. Susceptibility in maternal tissues positively correlates with the number of concepti, suggesting important contributions by expanded placental-fetal target tissue. Remarkably, these pregnancy-induced susceptibility phenotypes are also efficiently overturned in mice with resolved sublethal infection prior to pregnancy. Preconceptual infection primes the accumulation of E. coli-specific IgG and IgM antibodies, and adoptive transfer of serum containing these antibodies to naive recipient mice protects against fetal wastage. Together, these results suggest that the lack of E. coli immunity may help discriminate individuals at risk during pregnancy, and that overriding susceptibility to E. coli prenatal infection by preconceptual priming is a potential strategy for boosting immunity in this physiological window of vulnerability.

## INTRODUCTION

Pregnant women are uniquely susceptible to invasive systemic infection by a variety of classical prenatal pathogens, which can lead to congenital invasion and exceptionally high morbidity and mortality (1–6). An estimated half of early-preterm births (<28 weeks of gestation) and the majority of early-onset cases of neonatal sepsis are attributed to maternal infection during pregnancy (7–9). Maternal infection is also an important modifiable cause of stillbirth, especially in low- and middle-income countries (10–12). A recent prospective analysis of nearly 3 million live births across 52 countries shows that severe maternal infection occurs in 10.9 women per 1,000 live births and leads to disproportionately high rates of stillbirth and early neonatal death (13).

While pregnancy-induced immunological shifts have been probed primarily using pathogens such as Listeria monocytogenes, *Brucella* spp., and Zika virus, with established placental tropism and a unique predisposition for severe infection during pregnancy, it should also be highlighted that the more ubiquitous commensal pathobiont Escherichia coli is consistently the leading cause of maternal sepsis and bacteremia during pregnancy (14–18). For example, E. coli was the most common cause of maternal sepsis in a prospective analysis of 272 cases (>150,000 pregnancies) in Dublin, Ireland, between 2005 and 2012 (15). Likewise, E. coli was the most common cause of maternal bacteremia in a retrospective analysis of 347 cases (59,491 live births) in Paris, France, from 2005 to 2009 (16). E. coli is a Gram-negative bacterium that ubiquitously colonizes intestinal mucosal barrier tissues. It is also the most common cause of urinary tract infections and consistently a primary cause of bacteremia/sepsis in all age groups (19–24). The recent World Health Organization Global Maternal Sepsis study found the urinary tract to be the most common source of maternal infection and sepsis during pregnancy (13). Interestingly, while E. coli is consistently cited as one of the leading pathogens responsible for maternal sepsis during pregnancy, causing an estimated 33 to 50% of antenatal cases and 10 to 27% of fetal mortality (15, 16, 25, 26), the absolute risk associated with pregnancy has not been directly evaluated.

A number of pregnancy-associated physiologic shifts have been shown to promote susceptibility to infection by classical prenatal pathogens. For example, impaired tissue localization of innate immune cells and expansion of immunosuppressive maternal regulatory CD4^+^ T cells required for sustaining fetal tolerance increases the susceptibility of pregnant mice to infection by L. monocytogenes and Salmonella enterica serovar Typhimurium (27–29). Other studies show that the placenta is a nidus of infection in pregnant guinea pigs, responsible for efficient reseeding of maternal target tissues after systemic L. monocytogenes infection (30). For these classical prenatal bacterial pathogens, which reside and replicate primarily within host cells, transport within maternal leukocytes has been described to facilitate placental tropism and invasion of trophoblast cells (14, 31). In this context, while E. coli residence within bladder and vaginal epithelial cells and macrophage cells is increasingly recognized in the pathogenesis of urinary tract infections (32–37), key knowledge gaps remain regarding how pregnancy causes susceptibility to E. coli, which replicates primarily in extracellular tissue compartments (38, 39).

To investigate the immunopathogenesis of E. coli prenatal infection, a preclinical model employing pregnant mice was developed and shown to recapitulate the increased susceptibility of women to E. coli bacteremia during pregnancy. Using inbred mice with defined major histocompatibility complex (MHC) haplotype antigens housed under specific-pathogen-free conditions for mating and infection allows precise control over potentially important confounding factors, including maternal age, parity, maternal-fetal genetics, and prior pathogen exposures, so that the impacts of pregnancy on E. coli infection susceptibility can be addressed in isolation. This instructive model was used to further investigate the cause of maternal susceptibility to E. coli infection during pregnancy and to explore strategies for boosting immunity in this physiological context.

## RESULTS

### Pregnancy-induced susceptibility to systemic E. coli infection.

**FIG 1:**
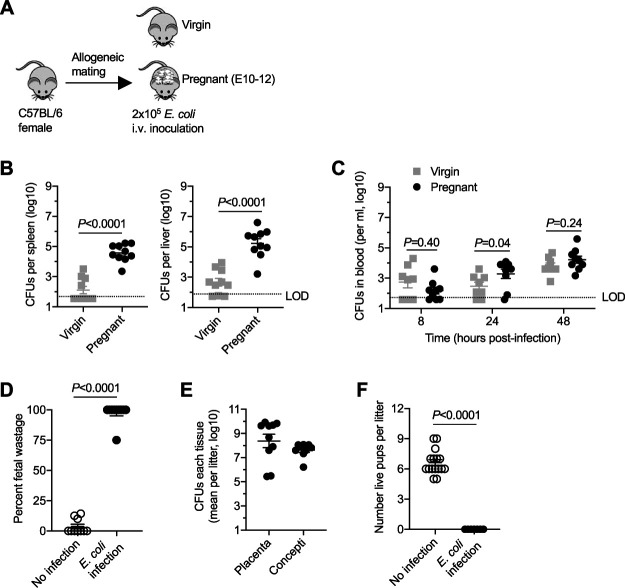
Pregnancy confers increased susceptibility to systemic E. coli infection in mice. (A) Schematic outlining the susceptibility to E. coli intravenous infection in virgin mice compared with mice midgestation (E10–12) during allogeneic pregnancy; (B) recoverable E. coli CFU in the spleen or liver 48 h after infection for the mice described in panel A; (C) E. coli CFU in the blood at each postinfection time point for the mice described in panel A; (D) percent fetal wastage among individual litters of mice 48 h after maternal E. coli infection at midgestation (E10–12) compared with that of no-infection control pregnant mice; (E) recoverable E. coli CFU in the placenta and concepti for each litter 48 h after maternal E. coli infection at midgestation; (F) number of live pups born at term among individual litters of mice 48 h after maternal E. coli infection at midgestation (E10–12) compared with that of no-infection control pregnant mice. Each point represents the data from an individual mouse, combined from at least two independent experiments, both with similar results. i.v., intravenous.

Pregnancy-induced shifts in E. coli infection susceptibility were initially evaluated by enumerating recoverable bacterial burdens in the target tissues of midgestation (embryonic day 10 to 12 [E10–12]) C57BL/6 (H-2^b^) mice and comparing them with those of isogeneic virgin C57BL/6 (H-2^b^) female control mice. The virulent uropathogenic E. coli strain UTI89 was utilized, since ascending infection from the urinary tract is a leading cause of maternal sepsis (35, 40), but for this study was administered intravenously so that pregnancy-induced changes in invasive infection susceptibility could be evaluated in isolation. Initial experiments used male mice in the BALB/c (H-2^d^) background to sire allogeneic pregnancy, which recapitulates the natural mismatch between maternal and fetal MHC haplotype antigens in humans and other naturally outbred populations (27, 41–43) (Fig. 1A). These experiments showed that pregnancy confers increased susceptibility to invasive systemic infection, with significantly increased (>100-fold) numbers of bacteria recovered from the spleen and liver 48 h after inoculation of midgestation (E10–12) pregnant mice compared with numbers in virgin control mice (Fig. 1B). E. coli was also recovered in the blood for a majority of mice after intravenous inoculation, with progressively increasing levels in the first 48 h and significantly higher numbers in pregnant than in virgin control mice 24 h postinfection (Fig. 1C). Thus, enhanced susceptibility to E. coli bacteremia during pregnancy is recapitulated in mice.

E. coli prenatal infection was also catastrophic with regard to pregnancy outcomes. Fifty percent (8 of 16) E. coli-infected pregnant mice showed vaginal bleeding within the first 48 h after infection, indicative of ensuing fetal complications. Necropsy revealed near complete (>95%) fetal wastage in the majority of E. coli-infected pregnant mice 48 h after maternal infection compared with background levels (<5%) in uninfected control pregnancies (Fig. 1D). E. coli was also consistently recovered in the placenta and concepti at remarkably high titers (>10^7^ CFU per tissue), highlighting the potential for efficient *in utero* fetal invasion (Fig. 1E). Extending this analysis to term showed complete loss of live pups in E. coli-infected pregnant mice (*n* = 7), compared with an average litter size of ∼6 to 7 live pups in uninfected control pregnancies (Fig. 1F). Together, these results show that mice effectively recapitulate human susceptibility to systemic E. coli infection during pregnancy, enabling an instructive opportunity to investigate the pregnancy-associated changes responsible for prenatal infection susceptibility and strategies for boosting antimicrobial immunity in this developmental window.

### Prenatal E. coli susceptibility linked with expanded fetal target tissue.

**FIG 2:**
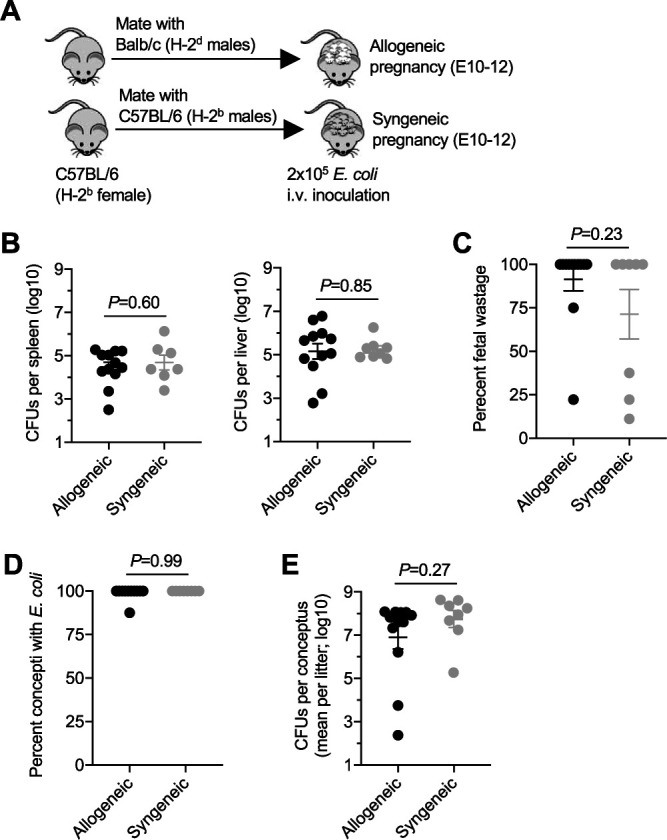
Susceptibilities to systemic E. coli infection are comparable during allogeneic and syngeneic pregnancies. (A) Schematic outlining the use of BALB/c (H-2^d^) or C57BL/6 (H-2^b^) males to establish allogeneic and syngeneic pregnancies, respectively, in C57BL/6 (H-2^b^) female mice; (B) recoverable E. coli CFU in the spleen or liver 48 h after infection at midgestation (E10–12) for the mice described in panel A; (C) percent fetal wastage among individual litters of mice 48 h after maternal E. coli infection at midgestation for the mice described in panel A; (D) percent concepti with recoverable E. coli CFU 48 h after maternal E. coli infection at midgestation for the mice described in panel A; (E) average number of recoverable E. coli CFU among concepti in each litter 48 h after maternal E. coli infection at midgestation for the mice described in panel A. Each point represents the data from an individual mouse, combined from at least two independent experiments with similar results.

Pregnancy stimulates a variety of immunological changes both systemically and locally at the maternal-fetal interface to avert rejection of semiallogeneic fetal tissues (44–46). The magnitude of these immunological changes, including expansion of immune-suppressive FOXP3^+^ regulatory CD4^+^ T cells, is directly proportional to the degree of antigenic mismatch between maternal and fetal tissues and drives susceptibility to some prenatal pathogens, including L. monocytogenes and *Salmonella* spp. (27, 29, 47). To investigate the contribution of maternal immunological changes driven by mismatch between maternal and fetal MHC haplotype antigens, E. coli infection susceptibility was evaluated in C57BL/6 (H-2^b^) female mice bearing allogeneic pregnancies (sired by BALB/c H-2^d^ male mice) and compared with that of mice with syngeneic pregnancies (sired by C57BL/6 H-2^b^ male mice) (Fig. 2A). We reasoned that if immunological shifts required for sustaining fetal tolerance play dominant roles conferring prenatal susceptibility during allogeneic pregnancy, susceptibility would be significantly reduced in syngeneic pregnancies. Surprisingly and in sharp contrast to this hypothesis, similar levels of susceptibility to prenatal E. coli infection were found in all groups of pregnant mice. This includes indistinguishably high levels of recoverable E. coli CFU in maternal tissues (spleen and liver) (Fig. 2B), fetal wastage (Fig. 2C), congenital fetal invasion (Fig. 2D), and recoverable E. coli CFU in each conceptus (Fig. 2E) of mice bearing allogeneic and syngeneic pregnancies. Thus, prenatal susceptibility to E. coli infection occurs regardless of immunological adaptations stimulated by mismatch between maternal-fetal MHC haplotype antigens.

**FIG 3:**
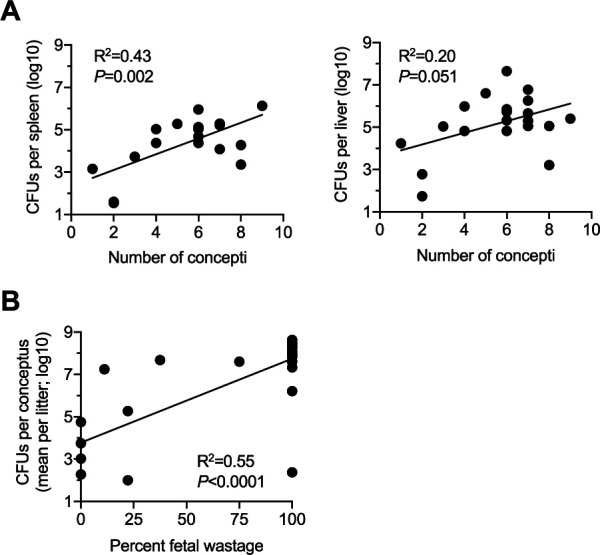
Maternal E. coli susceptibility during pregnancy directly correlates with the number of concepti in each litter. (A) Regression analysis comparing the number of concepti in each litter with recoverable E. coli in the maternal spleen and liver; (B) regression analysis comparing fetal wastage with the average number of recoverable E. coli CFU in the concepti of each litter. Each point represents the data from an individual mouse, combined from at least two independent experiments with similar results.

An alternative explanation for enhanced infection susceptibility during pregnancy is the presence of expanded placental-fetal target tissue that is susceptible to microbial invasion, as was shown for prenatal pathogens with placental tropism (30). This possibility was evaluated using the aforementioned mice infected with E. coli during pregnancy by comparing the relationship between susceptibility in maternal and fetal tissues and the natural variation in the number of concepti per litter. This analysis showed highly significant positive correlations between E. coli bacterial burden in the maternal spleen and the number of concepti in each litter (*P = *0.002) (Fig. 3A). Pregnant mice containing the highest E. coli bacterial burden in the maternal spleen also contained the most concepti, whereas the E. coli bacterial burden in maternal tissues progressively declined in pregnant mice with smaller numbers of concepti (Fig. 3A). Similar positive correlations were also found between E. coli bacterial burden in the maternal liver and the number of concepti in each litter; these border on statistical significance (*P = *0.051) (Fig. 3A). This potential causative link associated with expanded placental-fetal target tissue driving E. coli prenatal infection susceptibility extends to pregnancy outcomes where a direct correlation between percent fetal wastage and the average number of recoverable E. coli CFU in each conceptus per litter was identified (Fig. 3B). Together, these results suggest that maternal E. coli infection susceptibility and ensuing pregnancy complications are driven primarily by bacterial replication in expanded placental-fetal target tissue, with noncontributory roles for immunological changes stimulated by maternal-fetal antigenic mismatch.

### Preconceptual priming overrides prenatal E. coli infection susceptibility.

Immunity primed by E. coli systemic infection against recurrent infection remains poorly defined. The estimated 10 to 15% rate of recurrent E. coli bacteremia among individuals with prior bloodstream infection suggests incomplete immunity primed by natural infection (48–52). However, the vast majority of recurrent infections occur in immunocompromised individuals or individuals with other infection risk factors, including the presence of intravascular catheters, prosthetic tissues, or other implantable hardware (50, 52–55). Thus, immunity from prior infection may play a more dominant protective role in healthy, immunocompetent individuals, but this is difficult to determine given the retrospective nature of most human studies and the preponderance of underlying conditions in bacteremic patients. We reasoned that establishing whether E. coli infection primes protection against recurrent systemic infection and the potential persistence of these protective effects during pregnancy would create an instructive framework for developing vaccines to mitigate prenatal infection susceptibility.

**FIG 4:**
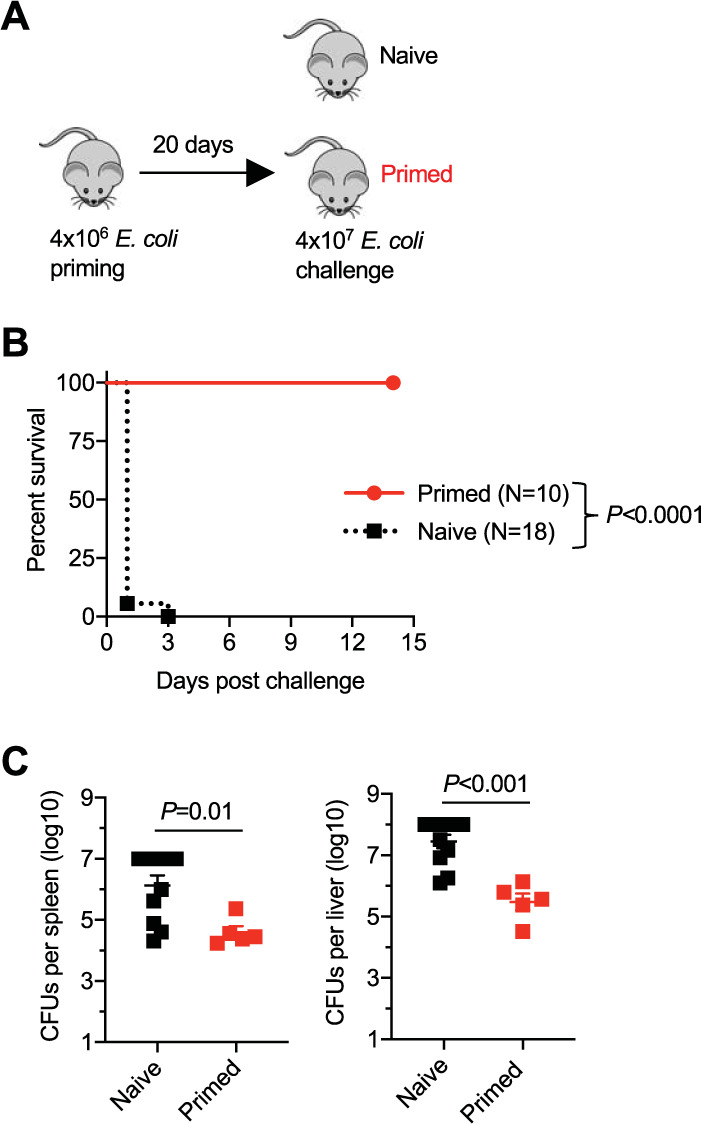
E. coli infection primes protective immunity against reinfection in mice. (A) Schematic outlining the susceptibility to E. coli high-dose (4 × 10^7^ CFU) challenge of specific-pathogen-free naive mice compared with that of mice infected 20 days prior with a sublethal E. coli inoculum; (B) survival for each group of mice described in panel A after high-dose E. coli challenge; (C) recoverable E. coli CFU in the spleen or liver 24 h after high-dose E. coli challenge for the mice described in panel A. Each point represents the data from an individual mouse, combined from at least two independent experiments with similar results.

These hypotheses were investigated by first evaluating the impacts of prior E. coli infection on susceptibility to reinfection with the same strain in nonpregnant mice. Initial dose titration experiments showed that 4 × 10^6^ E. coli CFU was uniformly nonlethal and cleared within 10 days postinfection, whereas 4 × 10^7^ CFU caused near complete mortality within the first 24 to 48 h postinfection and was associated with very high numbers of recoverable bacteria in the spleen and liver. An experimental framework using these inocula for E. coli priming and challenge, respectively, showed susceptibility to be sharply reduced in mice during secondary infection compared with that during primary infection (Fig. 4A). No mortality occurred after E. coli challenge in mice with a prior resolved sublethal infection (*n* = 10), compared with the rapid progression to a moribund state in control mice without a prior E. coli infection (Fig. 4B). The improved survival of mice with prior E. coli priming also paralleled significantly reduced E. coli bacterial burdens in the spleen and liver after high-dose challenge compared with those of control mice without prior E. coli infection (Fig. 4C). Thus, primary E. coli bacteremia efficiently primes cross-protection against recurrent systemic infection by the same strain.

**FIG 5:**
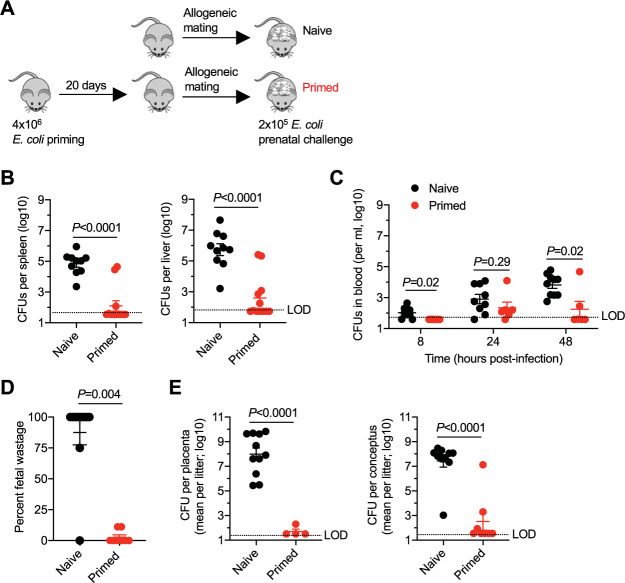
Preconceptual infection overrides pregnancy-induced E. coli infection susceptibility in mice. (A) Schematic outlining the susceptibility to E. coli prenatal challenge in specific-pathogen-free naive mice compared with mice infected 20 days prior to mating to establish allogeneic pregnancy; (B) recoverable E. coli CFU in the spleen or liver 48 h after infection for the mice described in panel A; (C) E. coli CFU in the blood at each postinfection time point for the mice described in panel A; (D) percent fetal wastage 48 h after maternal E. coli infection at midgestation (E10–12) among E. coli-naive (no preconceptual infection) and primed female mice with resolved E. coli infection prior to pregnancy; (E) recoverable E. coli CFU in the placenta and concepti for each litter 48 h after maternal E. coli infection at midgestation. Each point represents the data from an individual mouse, combined from at least two independent experiments with similar results.

The scope of these experiments was expanded to further investigate whether the protective benefits primed by resolved E. coli infection are sustained during pregnancy (Fig. 5A). We found that susceptibility to prenatal E. coli infection was sharply reduced in mice with resolved infection prior to mating compared with that of pregnant mice without prior E. coli priming. After E. coli challenge at midgestation (E10–12), bacterial burdens in the maternal spleen, liver, and blood were each significantly reduced in mice with resolved preconceptual infection compared with those of control mice without prior E. coli infection (Fig. 5B and C). The frequency of fetal wastage was also reduced to only background levels after E. coli prenatal infection in mice with prior resolved E. coli infection before pregnancy compared with the near uniform fetal wastage in E. coli naive pregnant control mice (Fig. 5D). Likewise, numbers of recoverable bacteria in the placenta and concepti were significantly reduced and below the limits of detection after E. coli infection for a majority of mice with resolved preconceptual infection compared with numbers in control mice without prior E. coli infection (Fig. 5E). Together, these results show that protective immunity primed by resolved E. coli infection is functionally retained during pregnancy.

**FIG 6:**
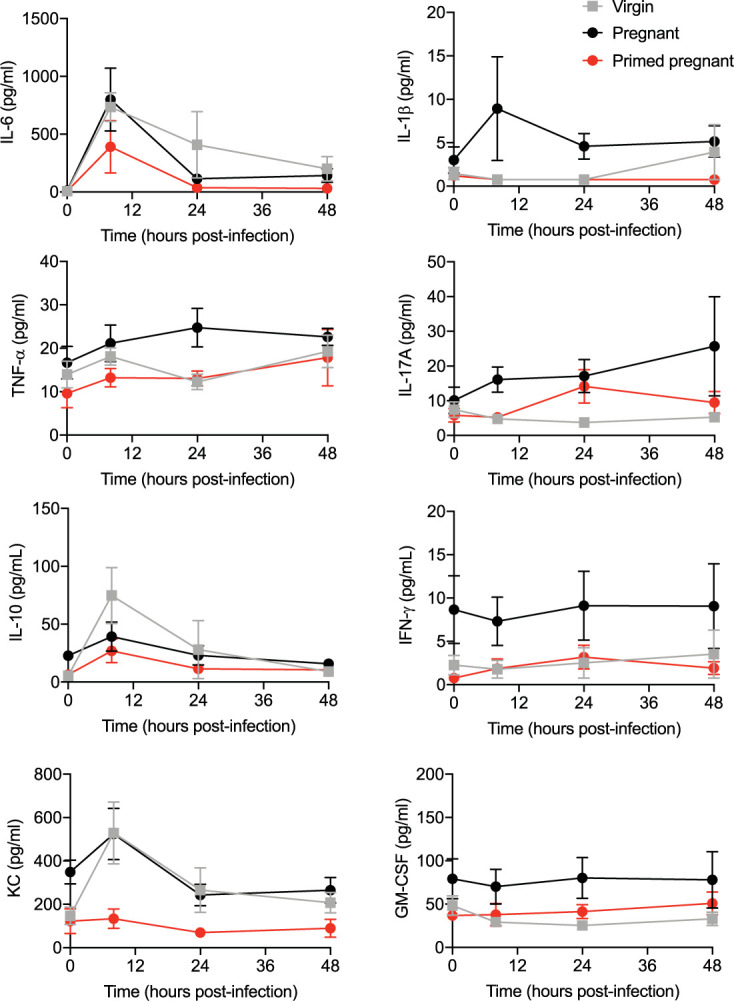
Serum cytokine levels after E. coli prenatal infection. Levels of each cytokine in the serum prior to or 8, 24, or 48 h after intravenous injection of E. coli (2 × 10^5^ CFU of uropathogenic strain UTI89) for virgin control mice (gray squares), pregnant midgestation (E10–12) mice without prior E. coli exposure (black circles), and pregnant midgestation (E10–12) mice with resolved E. coli infection prior to pregnancy (red circles). These data are representative of 4 to 5 mice per group at each time point, combined from two independent experiments with similar results.

E. coli bacteremia is highly inflammatory, and infection often triggers a cascade of innate proinflammatory cytokines, including tumor necrosis factor alpha (TNF-α), interleukin 1 (IL-1), and IL-6, which are both protective against infection and promote the immune pathogenesis of clinical sepsis (56–59). In turn, dynamic changes in the levels of these cytokines also occur with the progression of pregnancy, and perturbations have been implicated in various pregnancy complications, including preeclampsia and miscarriage (60–64). To evaluate pregnancy-induced shifts in E. coli infection susceptibility, serum levels for a variety of innate inflammatory cytokines were analyzed in the first 48 h after E. coli infection in pregnant and virgin control mice and between susceptible pregnant mice and more resistant pregnant mice with preconceptual E. coli priming. As expected, the levels of IL-6, TNF-α, IL-10, and the neutrophil chemoattractant keratinocyte-derived chemokine (KC) were increased in the sera of virgin control mice after E. coli infection (Fig. 6). Interestingly, the levels and accumulation tempos for some cytokines were nearly indistinguishable between E. coli-naive pregnant and virgin control mice despite drastic differences in tissue pathogen burden (Fig. 1). In particular, levels of IL-6 and KC peaked to similar levels within the first 8 h, later declining to background levels 48 after infection in both groups of mice (Fig. 6). In contrast, other cytokines showed more significant differences after E. coli infection between pregnant and virgin control mice. For example, infection-induced levels of TNF-α, IL-1β, and IL-17A were higher in the sera of pregnant mice than in the sera of virgin control mice, which parallels their increased bacterial burden, whereas the anti-inflammatory cytokine IL-10 peaked to higher levels in virgin than in pregnant mice (Fig. 6). Interestingly, levels of other cytokines, such as gamma interferon (IFN-γ) and granulocyte macrophage colony-stimulating factor (GM-CSF) did not change significantly after E. coli infection in virgin control or pregnant mice. Importantly, the production of nearly all cytokines was muted after E. coli infection of pregnant mice with resolved subclinical infection prior to mating, compared with that of pregnant mice without prior E. coli exposure (Fig. 6). Thus, pregnancy-induced susceptibility to sharply increased E. coli accumulation in target tissues, fetal wastage, and infection-induced inflammation are efficiently averted by preconceptual priming.

### E. coli-specific antibodies primed by primary infection protect against secondary challenge.

**FIG 7:**
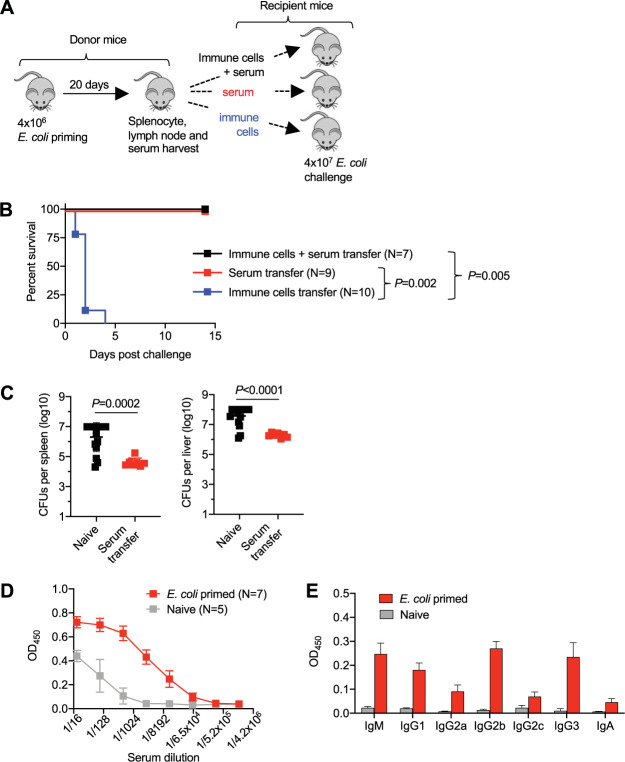
Serum containing E. coli-specific antibodies from mice with resolved infection transfers protection to naive recipient mice. (A) Schematic outlining when immune cells (splenocyte and lymph node cells) and serum (200 μl after heat inactivation) are harvested from E. coli-primed donor mice and transferred (5 × 10^7^ splenocytes plus lymph node cells and/or 200 μl heat inactivation serum) to each group of naive recipient mice; (B) survival for each group of mice described in panel A after high-dose (4 × 10^7^ CFU) E. coli challenge; (C) recoverable E. coli CFU in the spleen or liver 24 h after high-dose E. coli challenge for mice administered serum 1 day prior to infection compared with that in naive control mice given no serum; (D) E. coli-specific IgG antibody titers in the sera of E. coli-primed mice (20 days after infection) compared with those of naive control mice; (E) optical density of each antibody type with E. coli specificity after administration of a 1:2,000 dilution of the serum from E. coli-primed mice (20 days after infection) compared with that of naive control mice. Each point represents the data from an individual mouse, combined from at least two independent experiments with similar results.

To investigate which adaptive immune components stimulated by E. coli primary infection mediate protection against secondary challenge, the susceptibility of naive mice receiving adoptively transferred donor leukocytes (5 × 10^7^ spleen and lymph node cells), heat-inactivated serum (200 μl), or both from E. coli-primed mice 1 day prior to high-dose (4 × 10^7^ CFU) E. coli challenge was evaluated (Fig. 7A). We found that protection was mediated primarily by immune components in the serum, since naive mice receiving adoptively transferred serum only (*n* = 9) or serum plus spleen and lymph node cells (*n* = 7) from E. coli-primed mice showed no mortality, whereas all naive mice receiving only donor cells (*n* = 10) became moribund or died within the first 4 days after E. coli challenge (Fig. 7B). In turn, bacterial burden was sharply reduced after challenge of mice given serum from E. coli-primed mice compared with that of naive control mice (Fig. 7C) and to levels comparable with those in intact E. coli-primed mice (Fig. 4B).

These results showing protection against E. coli invasive infection transferred by serum is consistent with the ability of E. coli-specific antibodies to mediate protection against invasive E. coli infection in other infection contexts, including other rodent infection models (65–71). To further evaluate the priming and accumulation of protective antibodies after primary E. coli infection, levels of E. coli-specific antibodies in the sera of E. coli-primed mice were compared with those of naive control mice. These analyses showed 128- to 512-fold (6 to 8 additional 2-fold serum dilutions to achieve the same optical density [OD] reading)-increased titers of E. coli-specific antibodies in mice 20 days after primary infection (Fig. 7D). All antibody subtypes were significantly increased in the sera of E. coli-primed mice, with IgM, IgG1, IgG2b, and IgG3 showing the most prominent differences from those in naive control mice (Fig. 7E). Thus, protection against E. coli systemic infection primed by prior infection is associated with the accumulation of E. coli-specific antibodies in the serum, and serum containing E. coli-specific antibodies efficiently transfers protection against E. coli systemic infection to recipient naive mice.

**FIG 8:**
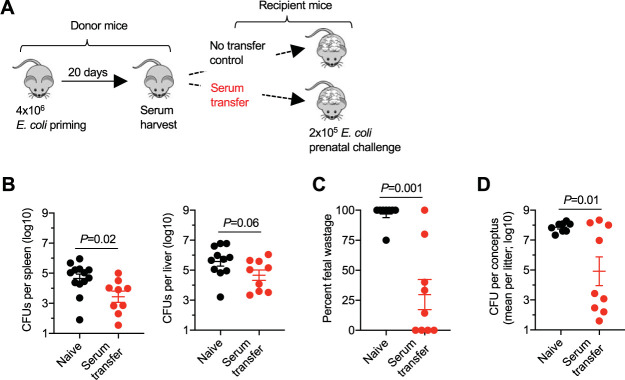
Serum containing E. coli-specific antibodies transfers protection to naive pregnant mice. (A) Schematic outlining when serum is harvested from E. coli-primed nonpregnant donor mice and transferred into pregnant mice midgestation (E10–12) during allogeneic pregnancy; (B) recoverable E. coli CFU in the spleen or liver 48 h after prenatal E. coli challenge for mice administered serum (200 μl after heat inactivation) 1 day prior to infection compared with that of naive control pregnant mice given no serum; (C) percent fetal wastage for the mice described in panels A and B; (D) average number of recoverable E. coli CFU among concepti in each litter 48 h after maternal E. coli prenatal challenge for the mice described in panels A and B. Each point represents the data from an individual mouse, combined from at least two independent experiments with similar results.

To investigate the efficiency with which donor immune serum containing E. coli-specific antibodies protects against prenatal E. coli infection, complementary experiments (i) used midgestation (E10–12) pregnant mice as recipients of serum from E. coli-primed mice and (ii) evaluated potential differences in maternal susceptibility to prenatal E. coli infection (Fig. 8A). We found that pregnant mice administered serum from E. coli-primed mice 1 day before E. coli infection during pregnancy contained significantly reduced bacterial burdens in the maternal spleen (*P = *0.02) and near significant reductions in the maternal liver (*P = *0.06) (Fig. 8B). Infection-induced fetal wastage and levels of E. coli in fetal tissues were also significantly reduced in pregnant mice given serum from E. coli-primed mice compared with those of control pregnant mice given no serum (Fig. 8C and D). Thus, circulating antibodies primed by prior invasive E. coli infection protects against reinfection, and these protective benefits persist during pregnancy, when infection susceptibility is naturally increased.

## DISCUSSION

Classical prenatal pathogens that cause more severe infection in mothers during pregnancy or have the propensity for congenital fetal invasion include Listeria monocytogenes, Toxoplasma gondii, *Brucella* spp., cytomegalovirus, and influenza and Zika viruses (6, 14, 31). This list should be expanded to include E. coli, which is consistently reported as a leading cause of maternal sepsis during pregnancy and responsible for an estimated 33 to 50% of antenatal cases (15, 16, 25, 26). Interestingly, however, the pregnancy-attributed risk of E. coli bacteremia has surprisingly not been described. Based on the reported incidence of E. coli bacteremia ranging from 64 to 100 cases per 100,000 pregnancies across multiple epidemiological surveys and the relatively low incidence in reproductive-age individuals (∼10 cases per 100,000 individuals 15 to 49 years of age) we conservatively estimate a 5- to 10-fold-increased risk during pregnancy (15, 16, 21, 22). This is likely an underestimation, since pregnancy status was not described in general surveys of E. coli bacteremia in reproductive-age individuals (aged 15 to 44 or 15 to 49 years) (21, 22), and a large proportion of these cases may be attributed to pregnancy. Thus, consideration of pregnancy as a biological variable is needed in human epidemiological surveys to more precisely define infection risk in this physiological context.

An intriguing commonality between classical prenatal pathogens is obligate or facultative residence within host cells and, except with influenza virus, defined placental-fetal tropism (6, 14). These parameters regarding whether microbes reside within or outside host cells during infection are likely linked with tissue tropism, since transport within circulating leukocytes promotes trophoblast cell invasion for intracellular prenatal pathogens (31). In turn, placental-fetal tropism promotes susceptibility to infection during pregnancy by intracellular pathogens, as infected placental-fetal cells can serve as a nidus for ongoing seeding of maternal tissues (30). We found that E. coli efficiently infects the placenta and other fetal tissues during systemic maternal infection, which may occur by several distinct mechanisms. These include the increasingly recognized ability of E. coli to replicate within infected host cells (such as macrophages and epithelial cells), which may promote placental-fetal invasion during prenatal infection, as with infection in the urinary tract (32–35, 37). Alternatively, extracellular pathogens like E. coli may exploit fundamentally different pathways, such as direct invasion of placental-fetal tissues from the maternal blood supply (72) or ascending spread from the female genital tract, where intracellular replication has also been identified (36).

Potential clues for dissociating these possibilities include the direct association between E. coli pathogen burden in maternal tissues and the number of concepti in each litter, and the high levels of E. coli in the placenta, suggesting expanded placental-fetal target tissue, directly contributes to prenatal E. coli infection susceptibility, as with intracellular pathogens like L. monocytogenes (30). However, an important distinction linked with the unique cellular residence of these pathogens lies in the adaptive immune components that mediate protective immunity. Primary L. monocytogenes infection confers protection against secondary challenge that is exclusively mediated by CD8^+^ T cells, consistent with the intracellular residence of the bacterium (73, 74). In contrast, donor splenocytes from E. coli-primed mice are nonprotective, and immunity against E. coli reinfection is instead mediated by antibodies, consistent with the primary extracellular residence of this bacterium (Fig. 7B). Nonetheless, we noted considerably increased variability in the levels of fetal wastage and E. coli burdens in fetal tissues among mice given serum from primed donors, in contrast to the near complete protection against fetal wastage and congenital fetal invasion observed when E. coli-primed mice were infected during pregnancy (compare Fig. 8C with Fig. 5D). The magnitude of protection in maternal tissues was also reduced considerably in pregnant mice administered serum from primed donors (∼10-fold reduction compared with that in controls) compared with that in E. coli-primed mice infected during pregnancy (∼1,000-fold reduction compared with that in controls) (compare Fig. 8B with Fig. 5B). This more attenuated protection with adoptive serum transfer in comparison to that of intact mice after primary infection most likely reflects that protective antibodies achieved with 200 μl donor immune serum is just at the threshold required for overriding heightened susceptibility during pregnancy.

In this context, it is important to also highlight pregnancy-induced changes in the frequency and tissue distribution of innate immune cells, including neutrophil and macrophage cells (28, 75, 76), both of which participate in protective immunity against E. coli and L. monocytogenes infections (38, 77–81). The cellular shifts that lead to infection susceptibility during pregnancy are likely linked with changes in systemic and local levels of innate inflammatory cytokines. For example, pregnant mice which rapidly succumbed to normally innocuous *Salmonella* infection were shown to have increased serum IL-6 levels, as well as blunted migration of leukocytes to infected target organs, whereas pregnancy-induced *Salmonella* susceptibility was reversed with IL-6 blockade (28). Interestingly, E. coli strains have drastically different potentials to evoke host proinflammatory cytokines after infection, and the uropathogenic E. coli strain UTI89 that we used has been shown to suppress inflammatory cytokine production after infection in other contexts (82). This may explain the muted production of some cytokines (IFN-γ and GM-CSF) that we observed after *in vivo* infection. Thus, further establishing the immunopathogenesis of prenatal E. coli infection will require complementary approaches, including mathematical modeling of the infection tempo after enumerating pathogen burden in maternal and fetal target tissues at multiple time points after infection (30), the use of E. coli strains recovered from women with systemic infections during pregnancy, the use of E. coli mutants that cannot enter and replicate within host cells for prenatal infection (34), and more precise analysis of systemic inflammation induced by infection within localized tissues (83).

Importantly, and despite these limitations, the enhanced susceptibility of women during pregnancy to systemic E. coli infection is replicated in specific-pathogen-free pregnant mice. Considering the ubiquitous presence of E. coli as a commensal pathobiont across mammalian species, these results raise important new questions as to why E. coli bacteremia with ensuing fetal complications does not occur even more frequently. In other words, if pregnancy confers susceptibility to invasive E. coli infection and E. coli is a ubiquitous pathobiont in the human intestine, would not infection during pregnancy be expected to be the norm and not the exception? We propose that there are likely yet-to-be-identified immunological or physiological distinctions unique to pregnant women who develop E. coli bacteremia. One consideration is the aforementioned discussion on expanded placental-fetal target tissue. This notion is consistent with the ∼5-fold-increased susceptibility to severe maternal sepsis during multiple gestations compared with that during singleton human pregnancies (84–86). A separate analysis of 29 pregnant women with E. coli bacteremia showed disproportionately increased susceptibility in the third pregnancy trimester (25). However, the majority of maternal E. coli bacteremia-sepsis cases occurring in singleton pregnancies, together with the lack of a clear association between susceptibility and the progression of pregnancy in larger studies (87), suggest that there are likely other factors, including the virulence of individual E. coli strains, immunity primed by prior infection or colonization, and maternal nutritional status (15–18). Likewise, prenatal susceptibility is also likely not driven by immunological changes required for accommodating the mismatch between expressed maternal and fetal MHC haplotype antigens, given the similar susceptibilities that we show between mice bearing syngeneic and those bearing allogeneic pregnancies.

Given the ubiquitous presence of E. coli in the intestinal lumen and the constant susceptibility to bloodstream seeding by these commensal pathobionts from mucosal interface tissue beginning early after birth (67), a provocative explanation for the relatively rare occurrence of E. coli bacteremia in human pregnancy is immunity naturally primed by subclinical infection in reproductive-age women prior to pregnancy. Using sublethal infection of specific-pathogen-free mice to mimic preconceptual exposure to invasive E. coli infection, we find nearly complete reversal of many parameters associated with prenatal susceptibility to E. coli infection. For example, recoverable E. coli in the maternal spleen and liver was significantly reduced to levels comparable to those in nonpregnant control mice with preconceptual priming (compare Fig. 1B and 5B). Likewise, near complete reversal of E. coli infection-induced fetal wastage was found in mice with resolved E. coli bacteremia prior to pregnancy (compare Fig. 1D and 5D).

Other limitations to our current model include using the same uropathogenic E. coli strain for priming and challenge, and the relatively short time interval between preconceptual priming and secondary prenatal challenge in these proof-of-concept experiments designed to probe pregnancy-induced shifts in host defense. Nonetheless, the dramatically reduced susceptibility to E. coli prenatal infection conferred by preconceptual priming also highlights interesting new strategies for potentially closing this developmental window of vulnerability. Recent studies show that natural antibodies primed by exposure to commensal bacteria have wide cross-reactivity against other Gram-negative *Enterobacteriaceae* species, including protection against enterotoxigenic E. coli infection primed by intestinal colonization with *Pantoea* spp. (67). Applied to the susceptibility of mothers to invasive E. coli infection during pregnancy, the larger translational implications are that natural antibodies primed by commensal E. coli or other cross-reactive *Enterobacteriaceae* spp. override in most women pregnancy-induced susceptibility to invasive infection. Screening for the presence of natural antibodies primed by the microbiota or subclinical invasive infection may help to discriminate women with natural immunity from those at increased risk for invasive E. coli infection during pregnancy. In turn, preconceptual administration of vaccines that mimic immunity primed by commensal colonization or subclinical invasive infection may efficiently override prenatal infection susceptibility. Beyond the susceptibility of mothers, the transfer of protective maternal antibodies *in utero* or through breastfeeding may also play dominant roles controlling the susceptibility of neonates to invasive E. coli infection (67). Important next steps include testing these hypotheses in preclinical models controlling for E. coli commensal colonization, and using urethral E. coli inoculation to better mimic the primary route of natural invasive infection.

## MATERIALS AND METHODS

### Mice.

Defined strains of inbred mice (C57BL/6 [MHC H-2^b^ haplotype] and BALB/c [MHC H-2^d^ haplotype]) mice were purchased from the National Cancer Institute and Charles River Laboratories (Frederick, Maryland) and maintained under specific-pathogen-free conditions at the Cincinnati Children’s Hospital. Allogeneic and syngeneic pregnancies in C57BL/6 female mice were sired by BALB/c and C57BL/6 male mice, respectively, as described previously (27). Experiments involving animals were performed under Cincinnati Children’s Hospital Institutional Animal Care and Use Committee (IACUC) approved protocols (assurance no. 2013-0170).

### Bacteria and infections.

For infection, E. coli strain UTI89 was grown in LB medium. Overnight cultures were back-diluted to log-phase growth (90 to 120 min, 37°C, 200 to 225 rpm; OD at 600 nm [OD_600_], 0.3 to 0.4). Thereafter, the bacteria were washed, resuspended, and diluted in sterile saline and injected via the lateral tail vein (in a 200-μl volume) into mice. For enumerating bacterial burden after infection, each tissue (spleen, liver, placentae, or concepti) was dissected in a sterile fashion from euthanized mice and homogenized in sterile saline supplemented with 0.05% Triton X-100. Serial dilutions of each tissue homogenate or heparinized blood was spread onto LB plates, and cells were counted after 24 h of incubation at 37°C.

### Passive serum and immune cell transfer.

Blood, spleen, and lymph nodes were harvested from virgin mice 20 days after E. coli priming (4 × 10^6^ CFU by intravenous infection). For serum harvest and transfer, the blood was allowed to clot at room temperature and then spun at 10,000 rpm for 10 min. Serum was removed and then heat inactivated (56°C for 20 min) and transferred by intraperitoneal injection into each group of recipient mice (200 μl) 1 day prior to E. coli infection. For immune cell harvest and transfer, the spleen and lymph nodes from donor mice were mechanically disrupted using frosted glass slides, lysed of red blood cells, filtered through a 60-μm nylon mesh, and resuspended in sterile saline. On average, 10^8^ splenocytes plus lymph node cells were recovered from each donor. Cells (5 × 10^7^; half donor mouse equivalent) were transferred by intravenous injection into each group of recipient mice 1 day prior to E. coli infection.

### Cytokine analysis.

At each time point after E. coli intravenous injection, blood was obtained from the retro-orbital space and allowed to clot at room temperature for each group of mice. The serum was harvested, frozen at −20°C, and analyzed using Milliplex (Millipore, Sigma).

### E. coli-specific antibodies.

For evaluating E. coli-specific antibodies by enzyme-linked immunosorbent assay (ELISA), flat-bottom, high-binding, 96-well enzyme immunoassay (EIA)/radioimmunoassay (RIA) plates (Costar) were coated with nearly confluent, log-phase E. coli UTI89 and allowed to dry overnight under UV light. E. coli-coated plates were blocked with 3% milk and first probed with serum dilutions from each mouse at the indicated dilution and then secondarily probed with the following biotin-conjugated anti-mouse antibodies: rat anti-mouse IgG (eBioscience catalog [cat.] no. 13-4013-8), rat anti-mouse IgM (eBioscience 13-5890-1589), rat anti-mouse IgA (eBioscience 13-5994-82), rat anti-mouse IgG1 (BD Pharmingen cat. no. 553441), rat anti-mouse IgG2a (BD Pharmingen 553388), rat anti-mouse IgG2b (BD Pharmingen 553393), rabbit anti-mouse IgG2c (Invitrogen cat. no. SA5-10235), and rat anti-mouse IgG3 (BD Pharmingen 553401). Each antibody was used at an 1:1,000 dilution and developed with streptavidin-peroxidase (554066; BD Bioscience) using *o*-phenylenediamine dihydrochloride as a substrate; absorbance at 450 nm (*A*_450_) was read as described previously (88).

### Quantification and statistical analysis.

The distribution of data on CFU, percent fetal invasion, and fetal wastage was first evaluated for a normal distribution. Thereafter, Student's *t* test and the nonparametric Mann-Whitney test were used for analysis of differences between normally and not normally distributed data sets. Linear regression was performed to determine correlations between E. coli bacterial burdens and the number of concepti per litter or the average E. coli bacterial burden per conceptus and fetal wastage in each litter. Survival between groups of mice was analyzed using the log rank (Mantel-Cox) test. All data were analyzed using GraphPad Prism software, and a *P *of<0.05 was taken as statistical significance.
